# Bioengineering Novel *in vitro* Co-culture Models That Represent the Human Intestinal Mucosa With Improved Caco-2 Structure and Barrier Function

**DOI:** 10.3389/fbioe.2020.00992

**Published:** 2020-08-31

**Authors:** Nicole J. Darling, Claire L. Mobbs, Ariana L. González-Hau, Matthew Freer, Stefan Przyborski

**Affiliations:** ^1^Department of Biosciences, Durham University, Durham, United Kingdom; ^2^Reprocell Europe Ltd, Sedgefield, United Kingdom

**Keywords:** Caco-2, fibroblast, 3D co-culture, epithelium, *in vitro*, intestinal, extracellular-matrix, human

## Abstract

The Caco-2 monolayer is the most widely used *in vitro* model of the human intestinal mucosa to study absorption. However, models lack communication from other cells present in the native intestine, such as signals from fibroblasts in the lamina propria. In this study, we have investigated the effects of fibroblasts upon the Caco-2 epithelium through two mechanisms: indirect signaling from fibroblasts and direct contact with fibroblasts. Culture of Caco-2 cells with paracrine signals from fibroblasts, through the use of conditioned media, did not induce a significant change in epithelial cell morphology or function. To examine the effects of direct contact between the epithelium and fibroblasts, we developed novel, humanized three-dimensional (3D) co-culture models whereby Caco-2 cells are grown on the surface of a subepithelial-like tissue construct containing intestinal or dermal fibroblasts. In our models, we observed endogenous extracellular matrix production from the fibroblasts that provides support to the above epithelium. The Caco-2 epithelium displayed morphological changes in 3D co-culture including enhanced polarization and the formation of a basement membrane-like attachment to the underlying stromal compartment. An important structural alteration was the significantly straightened lateral membrane that closely mimics the structure of the *in vivo* intestinal mucosa. This enhanced lateral membrane phenotype, in correlation with an reduction in TEER to levels more similar to the human intestine, is thought to be responsible for the increased paracellular permeability observed in 3D co-cultures. Our results demonstrate that direct contact between epithelial and mesenchymal cells results in an enhanced epithelial barrier. The *in vitro* models described herein have the potential to be used for studying intestinal epithelial-fibroblast interactions and could provide more accurate tools for drug permeability studies.

## Introduction

The study of intestinal function greatly relies on animal models. With an increasing demand to replace animal use in science, coupled with a lack of availability of *ex vivo* human tissue, *in vitro* intestinal alternatives are utilized to understand complex cellular processes such as drug-permeability and toxicity in a simplified format. The current gold-standard *in vitro* model used extensively throughout academia and industry for over 30 years is the Caco-2 monolayer. This Transwell^®^ -based culture system is one of the most extensively studied *in vitro* cell models due to its ability to form well-differentiated and polarized cell monolayers as surrogates for the human intestinal epithelium (Hidalgo et al., 1989). Although simple in design, Caco-2 monolayers possess important structural and functional characteristics of intestinal enterocytes, making them an attractive cell-line for investigating drug absorption. However, the models have many limitations which emanate from its adenocarcinoma origin and the simplicity of a monolayer culture system that lacks the complexity found in human tissue. For example, Caco-2 monolayers exhibit poor paracellular permeability to hydrophilic compounds as well as altered expression of efflux transporters, resulting in inaccuracy extrapolating *in vitro* permeability data to human tissue. Other limitations of the monolayer include abnormal cuboidal cell morphology as well as significantly heightened TEER compared to that of the normal human intestine.

Throughout the gastrointestinal tract there is a consistent quadruple layered structure comprising of a serosa, a muscularis propria, a submucosa and the innermost mucosa layer. The mucosa is defined as a layer of epithelial cells resting on a basement membrane supported by stromal cells. In the intestinal mucosa, the epithelial monolayer is situated above an extracellular matrix (ECM)-rich lamina propria, consisting of stromal cells, collagens, glycoproteins, and proteoglycans, separated by a protein-rich basement membrane. The ECM provides not only a physical scaffold for the cells but also mechanical and chemical signals fundamental to cellular processes (Kim et al., 2016). Epithelial-mesenchymal and epithelial-ECM interactions are essential for normal epithelial proliferation, differentiation and function (Dignass et al., 1994). Subepithelial fibroblasts directly influence the epithelial barrier through secretion of growth factors and cytokines, as well as indirectly through deposition of the ECM. The composition of the ECM affects enterocyte growth and function both *in vivo* and *in vitro* (Basson, 2001; Halttunen et al., 1996; Sanderson et al., 1996). Although the exact fibroblast secretome requires further elucidation, factors including transforming growth factor, keratinocyte growth factor, and epidermal growth factor are understood to influence the intestinal epithelial cells (Halttunen et al., 1996; Kim et al., 2012).

The most significant drawback of monolayer culture systems thus stems from the lack of complex, multicellular microenvironments that exist within the *in vivo* tissue. Recent models have sought to advance on these monolayer cultures either through the introduction of additional cell types or by using more complex tissue culture substrates. Most co-culture models include mucous secreting cells, immune components or fibroblasts, all of which have resulted in an enhanced epithelial layer (Béduneau et al., 2014; Pereira et al., 2015). One of the most common cell types added into Transwell^®^ co-cultures is the mucous-secreting cell line, HT29-MTX. Addition of these epithelial cells enables the influence of a mucous layer on the transport of compounds to be studied and it has been demonstrated that inclusion of these cells resulted in a higher correlation of passive permeability of hydrophilic compounds with the fraction absorbed in humans (Walter et al., 1996; Hilgendorf et al., 2000).

Stromal cells have also been included in co-culture models with Caco-2; mouse embryonic fibroblasts are frequently used to represent intestinal fibroblasts in these culture systems. Addition of fibroblasts in a collagen gel has been demonstrated to reduce TEER and alkaline phosphatase activity to more *in vivo*-like levels (Zhang et al., 2019). One of the most complex models has included Caco-2, HT29-MTX, mouse embryonic fibroblasts and immune components embedded within collagen gels and have shown an enhanced epithelial function with an increased absorptive permeability correlation to the human fraction absorbed (Li et al., 2013). However the fibroblasts used in these models are not of human origin and the stromal compartments lack in-depth characterization. Furthermore, these models lack the columnar and polarized epithelium like that of *in vivo* tissue. Although additional cell types enable a more accurate reconstitution of intestinal tissue, the use of animal-derived cells results in discrepancies when compared to the human intestine.

Further advances using the Transwell^®^ system have sought to use alternative culture substrates to support the growth of cells. Enhanced culture substrates most commonly utilize hydrogels, collagen-coated surfaces or nanofiber scaffolds, recapitulating *in vivo*-like structures (Holland-Cunz et al., 2004; Jabaji et al., 2014; Yi et al., 2017; Patient et al., 2019). As the most abundant ECM component, collagen I containing substrates in particular, successfully enhance enterocyte layer formation (Li et al., 2013; Jabaji et al., 2014). Collagen gels have also been adapted to recreate the villi structure observed in the intestine, leading to varying degrees of Caco-2 cell differentiation along the villus and increased permeability to hydrophilic compounds (Yu et al., 2012). HT29-MTX cells have also been included in such models and have the potential to be adapted to study bacterial attachment and invasion within the epithelium (Costello et al., 2014). Nevertheless, many of these gels remain poorly defined; they are mostly animal-derived, and display high variability. An additional concern with collagen gels is degradation, Caco-2 cells frequently invaded into degraded collagen structures resulting in the formation of structural abnormalities such as multilayers. Furthermore, some of the proteinaceous substrates commonly used consist of one or more ECM components, but also neglect other major ECM constituents such as fibronectin, which is implicated in epithelial attachment and integrity and is therefore essential in simulating the formation of *in vivo*-like tissue constructs (Kolachala et al., 2007).

In this paper, we have investigated the effect of two human fibroblast lineages of different tissue origins on the Caco-2 monolayer and have sought to determine whether secreted paracrine factors or direct contact with fibroblasts has greater effect on the epithelial structure and function. Herein, we present novel, three-dimensional human co-culture systems that more accurately simulate mucosal tissue layers representative of human intestine. These physiologically relevant, fully humanized mucosal constructs supplant previous models as they allow for abundant *in situ* ECM deposition by resident human fibroblasts, prominent basement membrane formation, as well as *in vivo* like epidermal lateral membrane morphology. Cell lines utilized for these models are routinely used and commercially available, making model reproducibility accessible in other laboratories. We hypothesize that the physical presence of fibroblasts co-cultured in 3D immediately adjacent to the epithelium will enhance enterocyte structure and function more than fibroblast conditioned media alone. Our intestinal models have been characterized structurally and functionally with direct comparison to human intestinal tissue. In doing so, we will investigate the effects of fibroblasts on enterocyte lateral membrane morphology: a phenomenon that until now, has not been previously reported in the literature. We propose that our humanized, robust and reproducible models will provide more accurate research tools for drug-permeability assays as well as the study of biological and biochemical processes in the intestinal mucosa. This relatively simple system overcomes the intricacies and expensive cell-culture expertise required by other *in vitro* models, such as organoid-based systems, whilst retaining the ability to closely reproduce features of the native human intestinal mucosa (Schweinlin et al., 2016).

## Materials and Methods

### Cell Maintenance

Caco-2 cells (861010202, ECACC, Porton Down, United Kingdom), CCD-18co (CRL-1459, ATCC, Middlesex, United Kingdom) and neonatal human dermal fibroblasts (HDFn, C0045C, Life Technologies, Fisher, Loughborough, United Kingdom) were maintained in T175 flasks (Greiner Bio-One, Kremsmunseter, Austria) in complete DMEM (Fisher), containing 10% fetal bovine serum (Fisher), 2 mM L-glutamine (Fisher) and 1% penicillin/streptomycin (Fisher) at 37°C, 5% CO_2_ and 95% humidity. Cells were passaged at 80% confluence using 0.25% Trypsin EDTA (Fisher).

### Transwell^®^ Cultures

Caco-2 cells were seeded at a density of 2.5 × 10^5^ onto Transwell^®^ polycarbonate filter supports (0.4 μm pore size, 12 mm diameter) (Fisher). Seeding density was optimized from previously published Caco-2 protocols that seeded 2.6 × 10^5^ cells per insert (Hubatsch et al., 2007). Cells cultured for a minimum of 21 days before analysis. Media was replaced every 2 days of culture with either fresh DMEM or fibroblast conditioned DMEM.

### Collection of Conditioned Media

Conditioned media was collected from both CCD-18co and HDFn fibroblasts. Once cells had grown to 80% confluence, fresh media was applied to the cells. After 48 h media samples were collected, centrifuged and sterile filtered through 0.2 μm filters to remove any cells or debris. Conditioned media was mixed 1:1 with fresh media before application to Transwell^®^ models.

### 3D Co-culture of Fibroblasts and Caco-2 Cells

To develop a viable co-culture model, robust foundations were first developed onto which Caco-2 cells were then seeded. 12-well Alvetex^®^ Scaffold inserts (Reprocell Europe, United Kingdom) were prepared according to manufacturer’s instructions. Fibroblasts were counted by the Trypan Blue exclusion assay and 0.5 × 10^6^ cells were seeded onto the inserts and were cultured in complete DMEM for 14 days with media changes every 3–4 days. HDFn cells were supplemented with 5 ng/mL TGF-β1 (Life Technologies) and 100 μg/mL ascorbic acid (Sigma-Aldrich, United Kingdom) to generate a sufficient cell population to allow the epithelial cells to be cultured on the surface. Additional supplements to the 3D culture of CCD-18co cells did not result in a confluent subepithelial compartment and instead, had an additional 0.5 × 10^6^ cells seeded at days 7 and 9 of culture to ensure sufficient fibroblasts were present within the scaffold. Caco-2 cells were then seeded onto 3D fibroblast cultures at a density of 0.4 × 10^6^ cells/insert and were maintained in complete DMEM without any supplements for a further 21 days.

### Paraffin Embedding and H&E Staining

*In vitro* models were washed in PBS prior to fixation in 4% paraformaldehyde (Fisher) for 2 h. Samples were dehydrated through a series of ethanols, followed by incubation in Histoclear (National Diagnostics, United States) then in 1:1 Histoclear:wax. Models were further incubated in wax before embedding and sectioning.

Paraffin sections were deparaffinized in Histoclear and rehydrated to dH_2_O before being stained in Mayer’s Hematoxylin (Sigma-Aldrich) for 5 min. Slides were then washed in dH_2_O and submerged in alkaline EtOH to blue the nuclei. Samples were dehydrated to 95% EtOH counter-stained in Eosin followed by dehydration to 100% EtOH. Slides were cleared twice in Histoclear and mounted in Omni-mount (National Diagnositcs) before imaging on a Leica microscope.

### Transmission Electron Microscopy

Tissue models were examined by transmission electron microscopy (TEM) by following standard procedures. Briefly, samples were fixed in Karnovsky’s fixative followed by post-fixation in 1% Osmium Tetroxide and ethanol dehydration and subsequently in Epon resin. Semi-thin (1 μm) sections were stained with toluidine blue and examined by light microscopy. Ultra-thin sections were stained with uranyl acetate and lead citrate and viewed under a Hitachi H7600 electron microscope.

### Scanning Electron Microscopy

Samples were fixed in Karnovsky fixative and osmium tetroxide and dehydrated through ethanol series as described for TEM sample preparation above. Samples were then critical point dried, attached onto silicon chips and sputter coated with platinum. Coated samples were imaged on a Hitachi S5200 field emission scanning electron microscope (SEM).

### Immunofluorescent Staining

Paraffin embedded samples were deparaffinized in Histoclear and rehydrated through 100%, 95 and 70% ethanol and PBS. Antigen retrieval was performed by incubation in citrate buffer at 95°C for 20 min. Samples were blocked in 20% newborn calf serum (Fisher) in 0.4% triton-X-100 PBS for 1 h. Primary antibodies (occludin sc-13256 and vimentin sc-6260 Santa Cruz Biotechnology; *E*-Cadherin #610181 BD biosciences; fibronectin ab23750; collagen I ab34710 and III ab7778 Abcam), diluted 1:100 in blocking buffer were added at concentrations recommended by the manufacture and incubated at 4°C overnight. Slides were washed x3 in PBS, incubated in secondary antibodies (donkey anti-mouse 488 A21202, donkey anti-rabbit 594 A21207, Invitrogen) diluted 1:1000 in blocking buffer for 1 h, washed again x3 in PBS before mounting in Vectashield with DAPI (Vector Labs, Peterborough, United Kingdom) and imaging on the Zeiss 880 confocal microscope.

### TEER Measurements

The integrity of the Caco-2 monolayer was determined by measuring the transepithelial electrical resistance (TEER) using an EVOM2 Voltohmeter with STX2 Chopstick probes. The final TEER value was determined by subtracting the TEER measurement from a blank Transwell^®^ insert and multiplying by the cell culture surface area (Equation 1):

(1) $$TEER\left(\Omega .cm^{2}\right)=\left(\Omega CellLayer-\Omega BlankInsert\right)\times I\,n\,s\,e\,r\,t\,S\,u\,r\,f\,a\,c\,e\,A\,r\,e\,a\,c\,m^{2}$$

### Permeability of Model Compounds Across the Epithelial Barriers

Transport studies were conducted in a standard Ussing chamber system (WPI, United Kingdom). Lucifer yellow (Sigma-Aldrich) was reconstituted in PBS (Sigma-Aldrich) and used at a final concentration of 100 μM. TEER was measured throughout the assay period to ensure continued model integrity throughout experimentation. Transport assays were conducted in HBSS, 2% Glucose *(v/v)* and maintained at 37°C by use of circulating water bath. Carbogen (95%, O_2_, 5% CO_2_,) (BOC, UK) was used to oxygenate assay solutions and circulate fluid across model epithelia. Drug compounds were added to the apical compartments (donor) at the beginning of the assay and samples were taken from the basolateral (receiver) compartment after 120 min. Apparent permeability of the models was calculated according to Equation 2:

(2) $$P\,a\,p\,p\,\left(c\,m/s\right)=\frac{V_{R}*d\,C_{R}}{d\,t*A*C_{D\,0}}$$

Where VR is the volume of the receiver compartment dC_*R*_/dt is the change in the analyte concentration of the receiver compartment over time, A is the area of the transport interface and C_*D*__0_ is the concentration of the donor compartment at time zero.

### Human Tissue Acquisition and Use

Fixed human intestinal tissues were collected by Biopta (Glasgow, United Kingdom) under appropriate ethical protocols in compliance with local laws and regulations. Tissues were received at Durham University under a formal MTA agreement and were processed following relevant UK HTA rules and guidelines at the time of publication.

### Statistical Analysis

All statistical analysis was done using Graphpad Prism 5 software by One-way ANOVA with Dunnetts post-test multiple comparison analysis using the Caco-2 monolayer on Transwell^®^ as the control. All values reported are averages ± SEM. The number of independent experiments in each instance (n) is noted in the figure legend.

## Results

### Construction of the Intestinal Models

**FIGURE 1:**
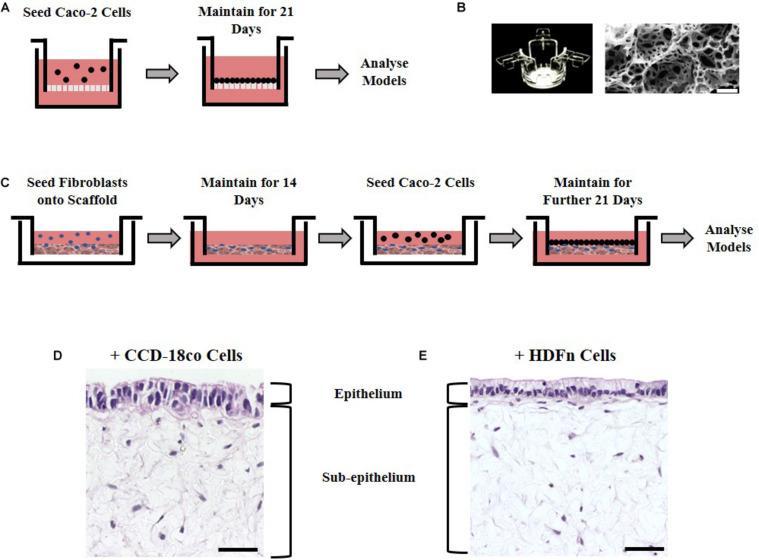
Experimental set up in building models of the intestine. **(A)** Schematic representation of Caco-2 cell culture methodology on Transwell^®^ inserts. **(B)** Alvetex^®^ well insert and scanning electron micrographs showing the structure of the Alvetex^®^ Scaffold membrane used in the co-culture models. **(C)** Schematic protocol depicting how fibroblast and epithelial cells were grown together in the 3D co-culture models. **(D)** H&E images of the resulting 3D co-culture models showing Caco-2 cells growing on top of Alvetex^®^ Scaffold populated with either one of the two types of fibroblasts tested: **(D)** CCD-18co; **(E)** HDFn. Scale bars: 50 μm.

Caco-2 cells are an extensively used cell line that can be differentiated into enterocyte-like cells of the small intestine when cultured for 21 days upon a porous Transwell^®^ polycarbonate membrane (Figure 1A). To investigate the effects of stromal cells on the structure and function of the epithelial cells, we employed two culture methods: paracrine factors from fibroblast conditioned media derived from 2D cultured fibroblasts or direct co-culture with fibroblasts grown in 3D. Two different fibroblast cell types of human origin were selected for this study: CCD-18co intestinal myofibroblast cells or HDFn skin fibroblasts. CCD-18co cells are one of the few commercially available intestinal fibroblast lineages and are thus widely used in the literature in co-cultures with Caco-2 cells that mimic the small intestinal function. They were selected as they are derived from the human colon and as such are a close match for Caco-2 cells which are also colonic in origin. Dermal fibroblasts are often used as a comparison to intestinal fibroblasts and their non-intestinal origin was used to test the importance of tissue-specific fibroblasts.

To examine the effects of fibroblast derived signals on the epithelium, conditioned media collected from either CCD-18co intestinal myofibroblast cells or HDFn skin fibroblasts and was mixed 1:1 with fresh media and applied to Transwell^®^ cultures every 2 days for 21 days. The second method was to allow the epithelial and stromal cells to have direct physical contact by developing a 3D co-culture model utilizing Alvetex^®^ Scaffold, an inert, porous polystyrene polyHIPE (Figure 1B). Fibroblast cells (CCD-18co or HDFn) were seeded onto the scaffold and were allowed to infiltrate the membrane for 14 days creating a robust foundation for Caco-2 growth. To achieve this, sequential seeding of CCD-18co cells was required as previously described while HDFn cells required additional supplementation of TGF and ascorbic acid. The overall outcome was the formation of a densely populated fibroblast compartment that could support the growth and differentiation of Caco-2 cells. Following the establishment of a 3D stomal cell component, Caco-2 cells were seeded onto the surface and cultured for a further 21 days to allow enterocyte differentiation to occur (Figure 1C); the resulting co-culture models show the scaffold populated with fibroblasts and an epithelial monolayer of Caco-2 cells on the surface in contact with the fibroblasts (Figures 1D,E).

### 3D Co-culture Systems Provide a More Structurally Relevant *in vitro* Model When Compared to Human Tissues and Transwell^®^ Controls

**FIGURE 2:**
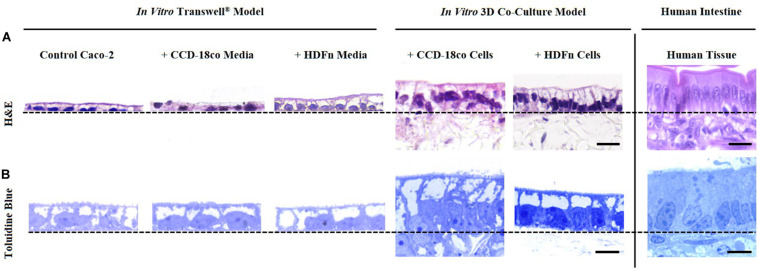
Morphological assessment of Caco-2 epithelia under alternate growth conditions. **(A)** H&E and **(B)** toluidine blue staining of control models where Caco-2 cells were cultured on either Transwell^®^ membranes alone; Transwell^®^ models cultured in conditioned medium from CCD-18co or HDFn fibroblasts; or Caco-2 cells in direct co-culture with the two types of fibroblasts on Alvetex^®^ Scaffold. Representative H&E and toluidine blue images of human small intestinal tissues are also shown for comparison. Note how the Caco-2 cell morphology changes and cells become more columnar, particularly in the 3D co-culture models. Scale bars: 20 μm **(A)** and 5 μm **(B)**.

Histological analysis of the *in vitro* models showed that Caco-2 cells form a monolayer of cells when cultured on Transwell^®^ membranes or on top of fibroblast cells (Figure 2). A brush-border can be observed through toluidine blue staining in all models indicating that the cells have differentiated into mature enterocytes with potential absorptive functions. Epithelial structural morphology did not significantly change with the addition of paracrine factors from fibroblast-conditioned media; however, cells show a significantly more elongated columnar phenotype when co-cultured with fibroblast cells in 3D culture. This enhanced polarization of the epithelium is more comparable to the *in vivo* intestine and is significantly altered compared to control Transwell^®^ -based models.

### Fibroblasts Provide Support to the Overlying Epithelium

**FIGURE 3:**
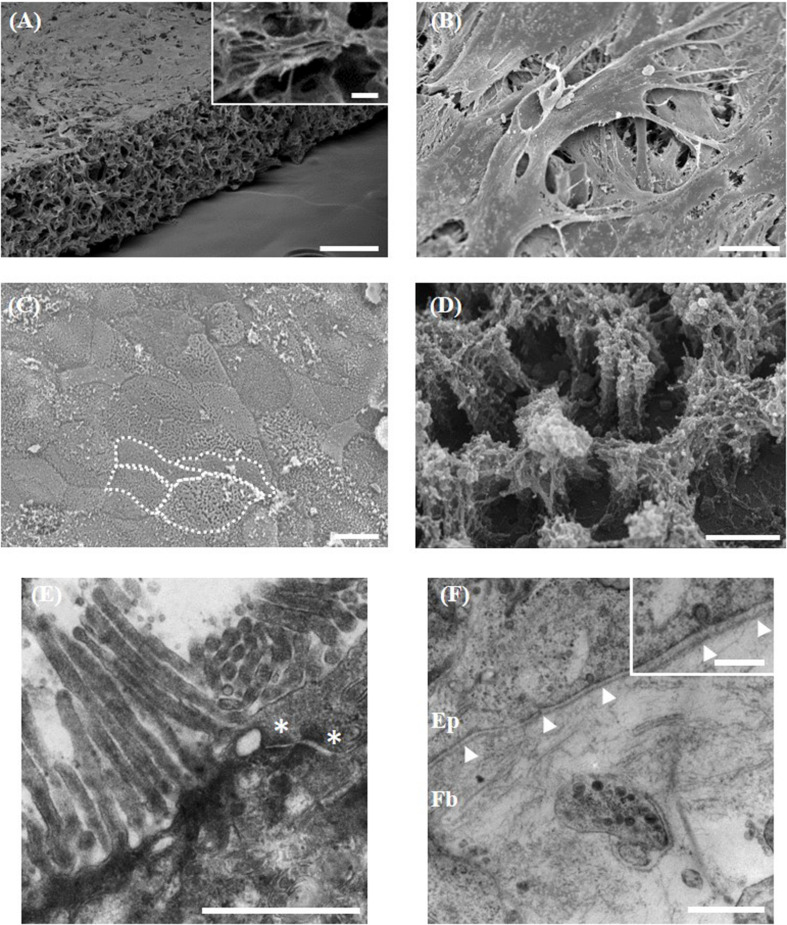
Morphological analysis of the 3D co-culture intestinal model using electron microscopy. **(A,B)** Representative SEM micrographs of the surface of the fibroblast compartment of the co-culture prior to seeding epithelial cells. (**A**, inset) Higher power imaging showing evidence of ECM deposition. **(C,D)** Surface of the Caco-2 monolayer (examples of individual cells outlined in white). **(D)** Higher power micrograph showing microvilli and glycocalyx. TEM micrographs of the: **(E)** microvilli brush-border and electron dense apical tight junctions (*); **(F)** the basement membrane (arrowheads) forming between the epithelial (Ep) cells and underlying fibroblasts (Fb). (**F**, inset) Higher power imaging showing vesicle transfer between epithelial cells and fibroblasts across the basement membrane. Scale bars: 200 μm **(A)**, 10 μm (**A**, inset), 20 μm **(B,C)**, 1 μm **(D–F)**, 250 nm (**F**, inset).

Direct co-culture of stromal and epithelial cells initially required the formation of a robust support onto which Caco-2 cells can grow. Fibroblasts populate throughout the Alvetex^®^ Scaffold membrane and build up on the surface of the scaffold (Figure 3A), generating the equivalent of a subepithelial compartment and acting as a foundation to provide structural support to the above enterocytes. Long-term differentiation of the fibroblasts in 3D (minimum of 14 days) is required to allow fibroblasts to layer on the surface of the membrane and to prevent epithelial cells infiltrating into the scaffold (Figure 3B). Culture of Caco-2 cells on Alvetex^®^ Scaffold without the support of the fibroblasts resulted in infiltration of the epithelium into the porous membrane and a loss of the monolayer structure (data not shown). Caco-2 cells formed a continuous monolayer when seeded on the surface of the 3D fibroblast culture. On the surface they exhibited a cobble stone morphology, typical of the apical pole of epithelial cells (Figure 3C), with clear evidence of microvilli associated with glycocalyx, a carbohydrate-rich coating over the surface of epithelial cells of the digestive tract (Figure 3D). TEM ultrastructural analysis highlights apical junctions in addition to electron dense layers at the base of the epithelial layer demonstrating the formation of a tri-lamina basement membrane between the epithelium and underlying fibroblasts and emphasizing the direct contact between the two cell populations (Figures 3E,F). No contact of Caco-2 enterocytes with the Alvetex^®^ Scaffold was found through TEM analysis. Cellular interaction between the fibroblasts and enterocytes appears evident under closer analysis, with clear vesicle transfer between the two cell populations (Figure 3F, inset).

### Fibroblasts Secrete Extracellular Matrix Proteins Within the Subepithelial Compartment

**FIGURE 4:**
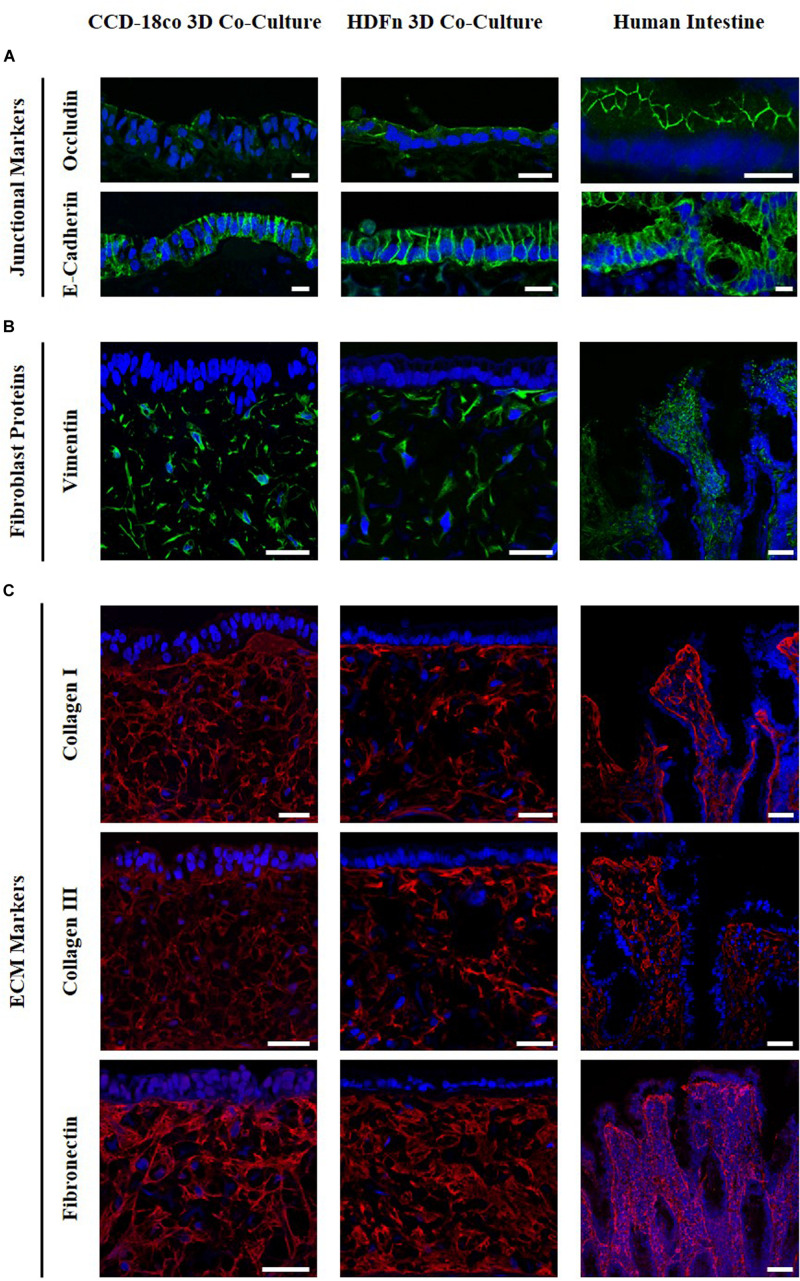
Comparison of 3D co-culture models with human intestinal tissue. Immunofluorescent staining of **(A)** the junctional proteins; occludin, *E*-Cadherin, **(B)** the fibroblast marker vimentin, and **(C)** collagens I and III and fibronectin counterstained with DAPI. Images show protein expression in Caco-2 co-culture models with intestinal fibroblasts (left) and human dermal fibroblasts (middle) compared to human intestinal tissues (right). Scale bar: 20 μm **(A)** and 50 μm **(B,C)**.

Immunofluorescent analysis (Figure 4) demonstrated that Caco-2 cells express the junctional markers occludin and *E*-cadherin, components of tight junctions and adherens junctions respectively. Occludin is present at the apical part of the cells while *E*-cadherin appears to be localized to the lateral membranes, in the same localization as in the intestine *in vivo.* Vimentin staining indicated the distribution of fibroblasts throughout the scaffold. Collagens I and III and fibronectin were found to be secreted by both intestinal and dermal fibroblasts, as well as being present in human intestinal tissue. Fibroblasts retain their metabolic activity throughout long term culture and continue to secrete ECM proteins (data not shown). The presence of both fibroblasts and ECM proteins is representative of a subepithelial tissue-like layer and results in the overall structure of the *in vitro* co-cultures being very similar to the *in vivo* intestine. Collectively, this acts as a foundation providing support for the intestinal epithelial cells.

### During 3D Co-culture Signals From Fibroblasts Induce a More *in vivo*-Like Epithelial Morphology

**FIGURE 5:**
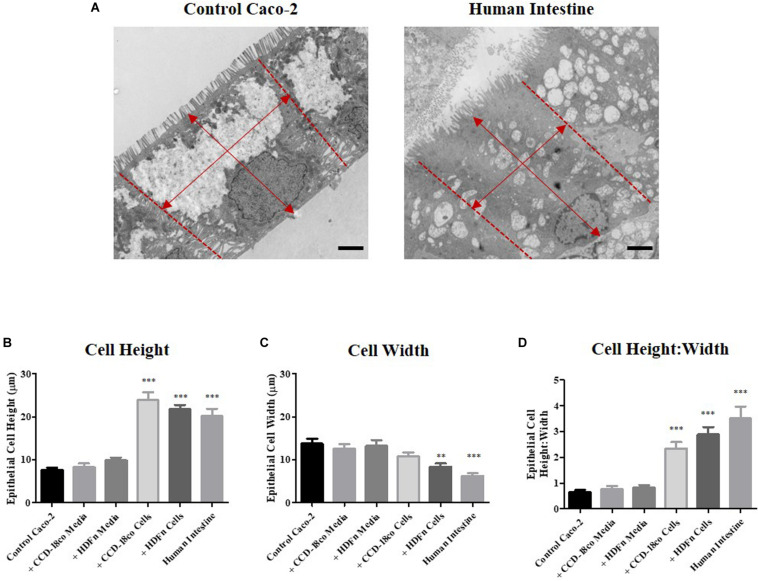
Morphometric analysis of epithelial cells in *in vitro* models and intestinal tissue. **(A)** Example TEM micrographs of Caco-2 Transwell^®^ control models and human tissue which were used to measure epithelial **(B)** cell height and **(C)** width [as indicated by arrows **(A)**], and **(D)** calculation of the ratio of epithelial cell height:width. Scale bars: 2 μm, *n* = 15 for *in vitro* models, *n* = 10 for human tissue, ^∗∗^*P* < 0.001, ^∗∗∗^*P* < 0.0001 compared to control Caco-2 monolayers.

Close examination of the ultra-structure through TEM analysis revealed that the height of Caco-2 cells increases when 3D co-cultured with fibroblasts. In contrast, this was not significantly altered by paracrine signals alone in conditioned media (Figure 5A). To determine whether the cells displayed a more cuboidal or columnar phenotype, we calculated the ratio of epithelial cell height:width; a ratio closer to 1 resembles a cuboidal phenotype while a number > 1 is indicative of a columnar cell, conversely a number < 1 suggests a flattened cell morphology. All Transwell^®^ -based models displayed a cuboidal phenotype, while 3D co-culture of Caco-2 cells with fibroblasts resulted in the cells becoming columnar in structure and more similar to the morphology of human intestinal epithelial cells (Figures 5B–D).

**FIGURE 6:**
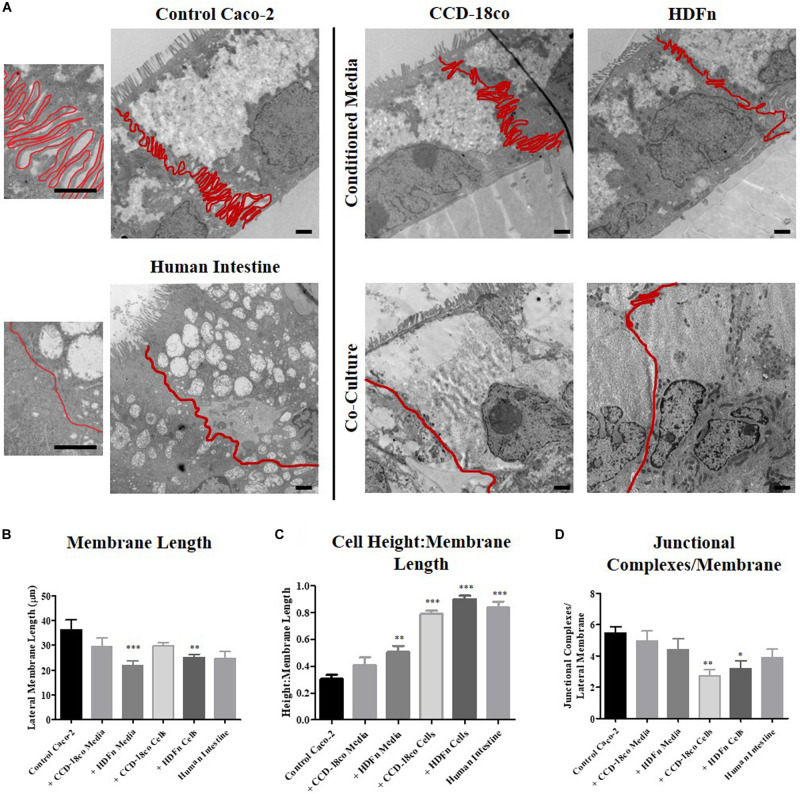
The profile and length of the lateral membrane is decreased in 3D co-culture models. **(A)** TEM images of control Caco-2 Transwell^®^ cultures; models cultured in fibroblast conditioned media; 3D co-cultured with fibroblasts; and human intestinal epithelial tissue. Lateral membranes of epithelial cells were traced and clearly outlined in red. Scale bars: 1 μm. **(B)** Lateral membrane length and **(C)** the ratio of cell height: membrane length of *in vitro* models compared to *in vivo* intestine. **(D)** Semi-quantitative analysis comparing the total number of electron dense junctions observed between adjacent epithelial cells. *n* = 15 for *in vitro* models, *n* = 10 for human tissue, ^∗^*P* < 0.01, ^∗∗^*P* < 0.001, ^∗∗∗^*P* < 0.0001 compared to control Caco-2 monolayers.

Analysis of the lateral membrane between adjacent epithelial cells was performed by carefully tracing the membrane on TEM images to determine its length (Figure 6A). Transwell^®^ Caco-2 models have a highly convoluted and interdigitated membrane, while Caco-2 cells co-cultured with fibroblasts in 3D and human tissue displayed a straightened membrane (Figure 6A). To directly compare lateral membrane length between cells in each of the *in vitro* models (Figure 6B), the length was normalized to epithelial cell height (Figure 6C). This demonstrated that conditioned media induces a slight change in the membrane structure that more closely resembles human tissue, and this change was significantly enhanced upon direct 3D co-culture of fibroblast and Caco-2 cells.

Quantitative assessment of the total number of electron dense junctional complexes per lateral membrane for each model type was determined (Figure 6D). Caco-2 control models on Transwell^®^ membranes had the highest number of junctions observed between adjacent cells. Fibroblast paracrine conditions did not significantly alter the number of junctional complexes between cells, but 3D co-culture reduced the number of electron dense complexes observed.

### Changes in Cell Structure Alter Epithelial Barrier Properties

**FIGURE 7:**
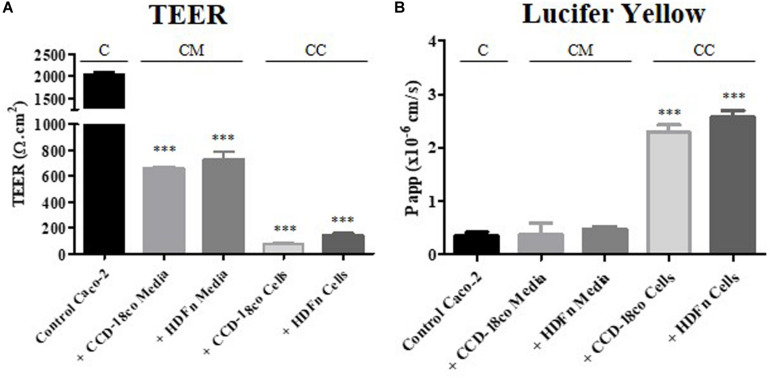
Functional assessment of the epithelial barrier properties for each *in vitro* model. **(A)** TEER was measured at day 21 of Caco-2 cell growth. **(B)** The apparent permeability co-efficient of the passive paracellular maker was calculated by performing transport assays on the models between days 21 and 25 of Caco-2 cell culture. “C” denotes the control Caco-2 monolayer, “CM” represents the conditioned media samples, while “CC” indicates the co-culture models. *n* = 3, ^∗∗∗^*P* < 0.0001 compared to control Caco-2 monolayers.

TEER measurements were taken at day 21 of Caco-2 culture once the cells had fully differentiated (Figure 7A). Caco-2 Transwell^®^ control models had a high TEER of over 2500 Ω.cm^2^, much higher than values reported for *in vivo* intestine (25–40 Ω.cm^2^) (Balimane and Chong, 2005). Conditioned media from fibroblasts, regardless of origin, reduced the TEER to 600–800 Ω.cm^2^ and this was further decreased below 200 Ω.cm^2^ when the Caco-2 epithelial layer was in direct 3D co-culture with the fibroblasts.

The concurrent decrease in TEER and straightened lateral membrane morphology was suggestive of a more permeable barrier formed by the epithelial cells. To investigate this further, we performed a simple transport assay with lucifer yellow, a marker that passes across the epithelium by passive paracellular transport, between the lateral membranes of adjacent cells (Figure 7B). Although TEER values were reduced when Caco-2 monolayers were treated with fibroblast paracrine factors, this was not reflected in the permeability of lucifer yellow, where no change in absorption was observed. However, in 3D co-culture, lucifer yellow permeability was increased almost 3-fold, correlating with the observed changes in TEER and the significantly straightened lateral membrane structure (Figure 7B).

## Discussion

The human adenocarcinoma cell line, Caco-2, forms the basis of many *in vitro* intestinal models as a result of their ease of culture and ability to form differentiated monolayers possessing enterocyte-like characteristics (Hidalgo et al., 1989). Despite these advantages, many limitations persist, in part preventing the replacement of animal and *ex vivo* human tissue models with Caco-2 cultures. The Caco-2 enterocyte layer exhibits structural and functional characteristics more like colonic tissue than small intestine, with higher TEER, lower permeability and lack of tissue complexity being the main differences (Balimane and Chong, 2005). In the present study, we propose novel, 3D models that overcome these limitations, whilst avoiding the many intricate and expensive steps associated with other *in vitro* intestinal models. Our intestinal tissue equivalents show enhanced epithelial structure and function more like that of native tissue, thus providing more suitable models for drug permeability studies as well as biological investigation of the mucosal layer.

In recent decades, scientists have endeavored to overcome the *in vitro* limitations of the Caco-2 Transwell^®^ system by using alternative culture substrates or through increasing complexity of models. Several advances have been made resulting in more physiologically relevant enterocyte monolayers with more realistic TEER and permeability values (Antunes et al., 2013; Béduneau et al., 2014; Pereira et al., 2015). However, such systems still lack endogenous molecules and interactions required to mimic the *in vivo* tissue dynamics. Some of these improved models include primary enterocytes or intestinal fibroblasts, both of which present issues of variability and lack of reproducibility (Takenaka et al., 2014; Schweinlin et al., 2016). Many co-culture systems also utilize fibroblast layers as a subepithelial compartment, which fail to account for the spatial organization within the mucosal tissue (Li et al., 2013; Matsusaki et al., 2015).

Organoid-based systems have recently appeared as the most likely frontrunner as they provide multicellular systems containing accurate cell niches. These cell culture systems contain all key intestinal epithelial cell types including enterocytes, goblet cells, enteroendocrine cells and Paneth calls; and have the unique ability to self-organize into 3D structures that recapitulate the crypt-villi structure observed *in vivo.* Organoids have the potential to be used for many different studies regarding the intestinal development process and disease modeling; additionally, they have been successfully engrafted into mice and show promise for regenerative medicine experimentation (Liu et al., 2016; Baumann, 2017; Cortez et al., 2018). However, these systems usually require costly media supplementation, including Wnt signaling pathway ligands and essential intestinal morphogens (Sato et al., 2011; Leushacke and Barker, 2014). Moreover, such systems are primarily epithelial only, they are effectively inside-out with the apical surface of the cells being completely enclosed in a central hollow region. Thus, their spatial arrangement greatly inhibits the use of organoids in the drug screening process (Yin et al., 2016; Costa and Ahluwalia, 2019). More recent advances have sought to seed organoids onto decellularised small intestinal submucosa (SIS) scaffolds which can shorten the differentiation time of the different intestinal cell types in addition to recreating the appropriate epithelial-ECM interactions. Use of SIS scaffolds more accurately recreates the intestinal mucosa anatomy and enables transport studies to be performed on the *in vitro* models (Schweinlin et al., 2016). Such systems, however, are not yet fully humanized and the expense, variability, and technical expertise required to culture also makes large-scale studies unfeasible. The technology described herein is robust and reproducible, using a standardized epithelial cell line and commercially available fibroblasts, enabling the relatively simple production of a multi-layered *in vitro* mucosal equivalent.

Substantial evidence exists suggesting intestinal epithelial structure and function are heavily influenced by cell-cell and cell-ECM interactions that are important in the complex microenvironment within the human mucosa (Halttunen et al., 1996; Kedinger et al., 1998; Bernardo and Fibbe, 2013). Fibroblast secreted peptides and extracellular matrix components have been shown to enhance epithelial morphology and function in a variety of tissues (Dignass et al., 1994; Korpos et al., 2010; Hausmann et al., 2019). Although fibroblast secretomes remain largely unknown, many peptide growth factors have been investigated using both primary cells and established cell lines (Halttunen et al., 1996; Hausmann et al., 2019). Among these, transforming growth factor-beta is a well-studied multifunctional cytokine postulated to be involved in epithelial regulation, immune homeostasis and enterocyte differentiation (Kojima et al., 1998; Ray et al., 2002). Furthermore, studies using CCD-18co and dermal fibroblast conditioned medium highlighted that fibroblast production of hepatocyte growth factor was partially responsible for enhanced proliferation of Caco-2 cells (Göke et al., 1998). Keratinocyte growth factor produced by both fibroblast types has also been implicated in enhancing the proliferation and differentiation of a range of different intestinal epithelial cells, including Caco-2 cells (Visco et al., 2009). Supplementary to this, CCD-18co and Caco-2 co-cultures highlighted the importance keratinocyte growth factor in altering tight junction protein expression with a subsequent decrease in TEER of monolayers (Kim et al., 2012).

It is reasonable to expect that fibroblasts from alternative tissue origins secrete different peptide factors and further work is necessary to elucidate these factors and understand the effects on Caco-2 enterocytes. What remains unclear, however, is the importance of fibroblast tissue origin on epithelial structure and function *in vitro*. In this study, we used two different fibroblast cell types in all of our investigations: a primary dermal fibroblast (HDFn) and a colonic cell line (CCD-18co). Both fibroblast lineages have previously used in co-culture studies with Caco-2 cells and have induced changes in the epithelium to better resemble the *in vivo* intestine. For example, co-culture of dermal fibroblast and Caco-2 cells induced a switch in the predominantly expressed carboxylesterase (CE) expression in Caco-2 cells from CE1 to higher CE2 expression more accurately reflecting the human small intestine (Matsusaki et al., 2015). Co-cultures developed using CCD-18co cells embedded in Matrigel have been shown to reduce TEER and P-gp expression and more accurately predicted the permeability of insulin than Transwell^®^ monolayers (Li et al., 2013; Pereira et al., 2015).

In our models, both fibroblast lineages tested supported the formation of a Caco-2 epithelium when co-cultured in 3D, with evidence of columnar cells. However, differences in epithelial morphology were obvious between Caco-2 cells grown in our 3D co-culture compared to models using fibroblast conditioned media alone, where epithelia were more like controls and were significantly more cuboidal in comparison. This suggests that fibroblasts in physical contact are more influential than the fibroblast secretome alone on the morphology of Caco-2 epithelial layers. This is most likely a result of the abundant endogenous ECM present in our 3D systems, as well as the formation of a supportive basement membrane. Fibroblasts are known to influence basement membrane composition, enabling more accurate cellular adhesion (Marinkovich et al., 1993). Accordingly, this is a significant advance over previous existing systems that utilize synthetic basement membrane equivalents (Patient et al., 2019). Positive staining for ECM components including collagens and fibronectin revealed the stromal-like tissue layer formed throughout the inert Alvetex^®^ Scaffold. Fibronectin in particular is an ECM component usually lacking in *in vitro* hydrogel and collagen coated models but is an extremely important protein in epithelial polarization (Kolachala et al., 2007). This *in situ* deposition of ECM by resident fibroblasts is a unique feature of our model that could prove invaluable in studies of epithelial-mucosal interactions and disease studies, particularly disorders involving ECM remodeling.

At present, our models only incorporate stromal cells and an absorptive epithelial cell line; inclusion of additional intestinal epithelial cell lineages, such as the HT29-MTX mucous secreting cell line could further enhance the structure and function of our models. HT29-MTX cells have been incorporated into Caco-2 co-cultures using scaffolds that recreate the hollow lumen structure of the intestine and the accumulation of mucous within these models induced a more physiologically relevant lower oxygen tension and allowed for the study of bacterial interactions (Chen et al., 2015). Reduced expression of the efflux transporter protein *P*-glycoprotein is also detected in these models resulting in more *in vivo*-like tissue constructs.

Lateral membrane interdigitation is a prominent characteristic of Caco-2 monolayers (Hidalgo et al., 1989). Previous studies have investigated the effect of fibroblast secreted factors and ECM components on enterocyte junctional complexes (Ichikawa-Tomikawa et al., 2011). However, to our knowledge, none have reported on the influence of fibroblasts on enterocyte lateral membrane morphology. Caco-2 cells cultured in the conventional Transwell^®^ system result in monolayers exhibiting an extremely interdigitated lateral membrane, a characteristic that could explain abnormally high TEER values and low permeability. Paracrine factors from both fibroblast cell types did not significantly decrease lateral membrane folding. In contrast, Caco-2 monolayers in 3D culture systems displayed significantly less lateral membrane interdigitation and lateral membranes appeared straighter which is characteristic of *in vivo* tissue. Supplementary to this, we found total junctional complexes per lateral membrane decreased in the 3D models compared to the standard Transwell^®^ model. These results suggest that epithelial-mesenchymal interactions are implicated in the structural connections between adjacent epithelial cells and lateral-membrane morphological dynamics. The lateral-basolateral membranes of enterocyte monolayers contain junctional complexes required for cellular adhesion and are implicated in paracellular transport.

Caco-2 cells are capable of spontaneous polarization. Varying degrees of polarization can be observed however, in different *in vitro* models (Gaillard and Finlay, 1996; Siccardi et al., 2004). Although all enterocyte monolayers exhibited polarization markers, such as brush border formation, those in our 3D co-culture models appeared more polarized than those grown in conditioned media and controls. Similarly, culture of Caco-2 cells on decellularised porcine jejunal segments induced an increase in cellular polarity, emphasizing the importance of epithelial-ECM interaction in *in vitro* models (Pusch et al., 2011). Decellularised small intestinal submucosa scaffolds are useful tools not only for recreating this crosstalk but also to induce the native intestinal architecture, which cannot currently be achieved in our 3D models. The decellularization process of such scaffolds, however, can result in ineffective ECM-remodeling subsequent to harsh decellularizing agents (Hussey et al., 2018). The establishment of polarity in enterocytes is important for barrier function and transport properties. Apical-basolateral polarity in many epithelial tissues can be enhanced through extracellular matrix components, particularly laminin (Hoffmann et al., 1996). This is a result of ECM interaction with enterocyte cell surface receptors such as integrins, which are known to affect polarity and junctional constituents such as *E*-cadherin in Caco-2 cells (Schreider et al., 2002). This is understood to be because cellular adhesions, both cell-cell and cell-ECM, connect to the cytoskeleton and thus influence the actin-dynamics involved in polarization. This could prove of particular interest in further experimentation, as integrin-mediated actin cytoskeletal changes may be the cause of the lateral membrane interdigitation reported herein. Further work is required to assess these interactions and actin cytoskeletons in our system. Whilst we might expect connections between cells to vary between different 3D co-culture models, interactions were considerably more *in vivo*-like compared to controls, most likely as a result of abundant ECM and basement membrane components.

We employed TEER to assess functional properties of the Caco-2 barriers in all of the culture systems. Conventional Caco-2 Transwell^®^ models are known to have extremely high TEER, unlike human tissue, and are also known to be extremely variable between different laboratories (Hidalgo et al., 1989). Many different factors affect TEER of Caco-2 monolayers including medium composition, culture period and temperature (Sambuy et al., 2005). Inter-lab variability in TEER has been demonstrated in a number of different studies that examined the effect of passage number on TEER; some groups demonstrated a decrease in TEER as cells age while others found conflicting results (Ranaldi et al., 1992; Yu et al., 1997). In the present study, Caco-2 TEER values were moderately decreased by paracrine-mediated mechanisms when cultured with conditioned media from both fibroblast cell types. The most profound decreases in TEER were observed in our 3D co-culture models; with values being much more similar to human small intestine *in vivo* suggesting that epithelial integrity is also significantly influenced by the subepithelial stromal compartment. Similarly, Caco-2 co-cultures with mouse embryonic fibroblasts in collagen gels have demonstrated a significant decrease in TEER from over 2000 Ω.cm^2^ in Caco-2 monocultures to levels below 1000 Ω.cm^2^ and demonstrated a concurrent increase in the apparent permeability of paracellularly transported compounds (Li et al., 2013; Zhang et al., 2019).

Examination of the permeability of lucifer yellow was performed to determine if there was any correlation between the level of lateral membrane interdigitation and paracellular permeability, as compounds may be retained for longer in the highly convoluted lateral membranes of Transwell^®^ monolayers. Increased paracellular permeability was observed in the 3D co-cultures, reflecting the decreased TEER. However, this was not the case in the Transwell^®^ -based paracrine systems which although demonstrated a reduction in TEER, retained abnormally low permeability values. It could therefore be implied that there is a correlation between degree of membrane interdigitation and paracellular permeability in enterocyte monolayers. Though beyond the scope of this study, further experiments are required to examine the effects of drug compound size on passive permeability and to determine of the effects of direct co-culture on the transcellular and efflux permeability.

## Conclusion

To summarize, we have developed novel 3D co-cultures recapitulating the intestinal mucosa. We showed that when co-cultured in 3D immediately adjacent to fibroblasts, Caco-2 monolayers display enhanced morphological characteristics such as enhanced columnar shape polarization and altered lateral membrane morphology more similar to *in vivo* enterocytes We used TEER and paracellular permeability to provide preliminary evidence that our 3D co-cultures were functionally enhanced, however, further experiments are required to demonstrate how the improved structure impacts the function of the enterocytes and to determine whether the 3D tissue constructs have a more *in vivo-*like function compared to controls. Such morphological and functional characteristics were not observed in Caco-2 cells grown in fibroblast-conditioned media alone, emphasizing that direct co-culture in 3D is more influential on Caco-2 epithelial structure and function than the fibroblast secretome alone. In conclusion, the 3D co-culture models presented herein produce more tissue-like enterocyte lateral membranes, a physiologically relevant attribute that could prove beneficial for the use of Caco-2 cells in permeability studies.

## Data Availability Statement

All datasets generated for this study are included in the article/supplementary material.

## Ethics Statement

The studies involving fixed human intestinal tissues were collected by REPROCELL Europe Limited (Glasgow, United Kingdom) under appropriate ethical protocols in compliance with local laws and regulations that were reviewed and approved by the West of Scotland Research Ethics Committee (REC Ref: 17/WS/0049). All tissue donors provided informed written consent. Tissues were received at Durham University under a formal MTA agreement and were processed following relevant UK HTA rules and guidelines at the time of publication.

## Author Contributions

AG-H, MF, CM, and ND performed the experiments. AG-H and MF conducted the data analysis. CM, ND, and SP wrote and revised the manuscript. SP had oversight of the project and approved the manuscript for submission. All authors contributed to the article and approved the submitted version.

## Conflict of Interest

SP is the original inventor of Alvetex^®^ technology now commercialized by Reprocell Europe Ltd. SP currently acts as a scientific consultant for Reprocell via a research collaboration with Durham University. CM is part of an ERDF sponsored studentship in collaboration with Reprocell Europe. The remaining authors declare that the research was conducted in the absence of any commercial or financial relationships that could be construed as a potential conflict of interest.
